# Prevalence of H. Pylori in Perforated Peptic Ulcer Disease at Saint Paul's Hospital Millennium Medical College, Addis Ababa, Ethiopia

**DOI:** 10.4314/ejhs.v31i5.8

**Published:** 2021-09

**Authors:** Mekdim Tadesse, Eyasu Musie, Berhanetsehay Teklewold, Endalkachew Hailu

**Affiliations:** 1 Department of Surgery, Saint Paul's Hospital Millennium Medical College, Addis Ababa, Ethiopia

**Keywords:** Perforated Peptic Ulcer Disease, H. Pylori eradication therapy, Saint Paul Hospital

## Abstract

**Background:**

Helicobacter Pylori is the most common cause of peptic ulcer disease with disputable association with perorated Peptic Ulcer disease (PPD). This study aims to determine magnitude of Helicobacter Pylori in PPD and the outcomes of treatment, at Saint Paul Hospital Millennium Medical College (SPHMMC)

**Method:**

Between January 9, 2013 and February 7, 2018, all patients operated for PPD were recruited retrospectively. Data was extracted from patient's medical records using pretested questionnaire. Data analysis was done by SPSS version 20

**Result:**

During the 5 years, 46 patients fulfilling inclusion criteria were included in the study. H. Pylori test was positive for stool antigen in 10 (21.7%) and serology eleven (23.9 %) of patients. Overall, nineteen (41.3%) of patients had positive result regardless of the type of test used. Out of 46 patients, twenty-six (56.5%) patients were given eradication therapy and thirty-four (73.9%) patients were given PPI alone or as a part of eradication therapy. During their hospital stay, five (10.9%) patients developed deep and superficial surgical site infection and two (4.3%) of patients have died.

**Conclusion:**

The prevalence of H. Pylori among PPD patients in this study is lower compared to most studies. Further prospective studies should be conducted in the future to understand association with H. Pylori and provide recommendations on eradication treatment.

## Introduction

Helicobacter Pylori (H. Pylori) is spiral-shaped gram-negative bacteria that is oxidase-positive, catalase positive and a strong producer of urease and plays an important role in the pathogenesis of peptic ulcer disease (PUD) (1,2). H. Pylori is associated poverty with an estimated prevalence of 70% living in LMIC compared to a maximum of 40% in developed countries, among which H. Pylori is positive in more than 95% with duodenal ulcer, and 70–80% with gastric ulcer (2, 3). Colonization rates exceed 70% in some groups and vary from less than 10% to more than 80% worldwide, in western countries the percentage of people with H. Pylori infection roughly matches age (i.e., 20% at age 20, 30% at age 30, and 80% at age 80 (3). H. Pylori is a major cause of chronic gastritis, it infects 50 % of the world's population one of its complications being perforation (5). Perforation occurs in 2–10% of PUD patients and account for more than 70% of deaths associated with PUD (6). Even though there is enough data regarding the relationship between uncomplicated PUD and H. Pylori infection (90–100%), data regarding Perforated Peptic Ulcer (PPD) and H. Pylori infection is limited (7). Several methods may be used to diagnose H. Pylori infection. These tests are: serological tests, urea breath test, endoscopy with biopsy (biopsy urease test, histologic identifications of organism, culture of biopsy specimen (8).

Studies in Ethiopia on the association of H. Pylori with uncomplicated PUD and outcomes on complicated PUD showed similar results as in other LMIC (2, 3, 8, 19, 21, 22). In our Hospital, PPD is among the commonest surgical emergencies that require immediate surgical intervention. However there are no studies on status of H. Pylori infection on PPD.

The aim of this study is to determine the H. Pylori status of patients with PPD so that decision makers will use the information for priority setting and make decisions based on existing evidence

## Material and Methods

Retrospective cross sectional study was done from January 1, 2019 to May 31, 2019; at SPHMMC on patients operated for perforated peptic ulcer disease (PPD) between January 9, 2013 and February 7, 2018 (five years period) .SPHMMC is a teaching referral hospital for patients from Addis Ababa and all over the country with catchment population of 6 million and performing 200–250 surgeries per month having surgical ward capacity of 300 beds. Department of surgery is one of the oldest departments in the college providing undergraduate, post graduate and fellowship programs staffed with more than 20 surgeons. The study included all patients operated for PPU during the study period that tested positive for the available H. Pylori tests and results are attached in the charts. Accordingly, out of the 141 patients operated for PPD during the five year period, the study was conducted on only 46 patients who had results of H. Pylori test attached to their chart.

Data collection was carried out by primary investigator from medical record, using pretested questionnaire on socio demographics, medical history, history of peptic ulcer disease, smoking, alcohol intake, use of NSAID, method of diagnosis utilized for H. Pylori and type of treatment given for H. Pylori, type of surgery performed for the PPU and the surgical outcome. Data was checked for completeness and analyzed using SPSS window version 20.0. A written Ethical Clearance letter of approval by the IRB of SPHMMC was obtained.

## Results

**Figure 1:**
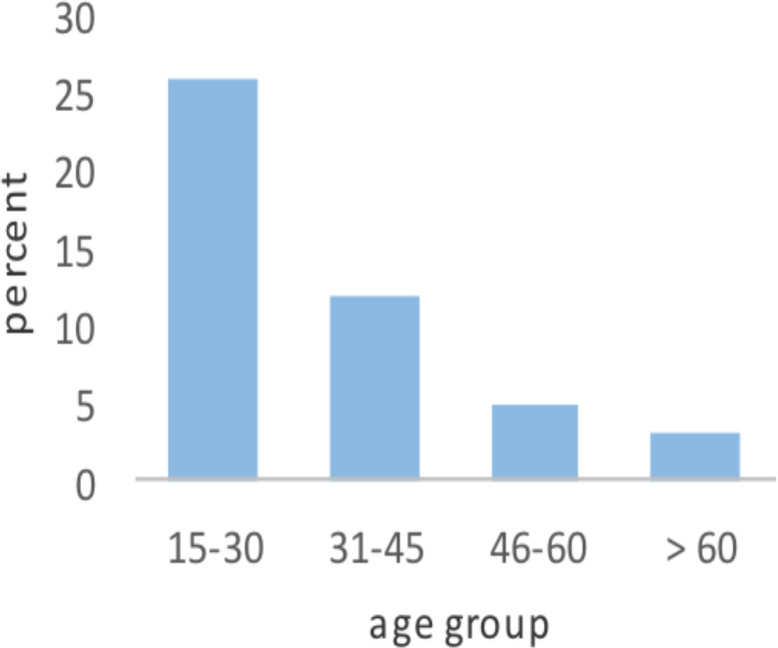
Age distribution of patients operated for perforated PUD at SPHMMC from January 9, 2013 to February 7, 2018.

**Socio-demographic characteristics**: Between January 9, 2013 and February 7, 2018, 141 patients were operated for PPU, and 46 patients were recruited for the study because of the exclusion criteria. Their mean age was 39.85 ± 16.12 SD with age range of 17–74 years (Figure 1). Thirty-nine (84.8%) were male. Out of 46 patients, five (10.9%), six (13 %), seven (15.2 %) patients were smokers, alcoholic, and chat chewers respectively.

**Clinical Presentation**: Twenty-four (52.2%) of patients presented less than 24 hours since the onset of abdominal pain; while Nine (19.6 %) patients presented after 72 hours after onset of the abdominal pain. Thirty-two (69.6%) of patients had history of chronic dyspepsia. None of the patients had documentation about history of use NSAIDs. For over half of the patients, twenty-eight (61%) other risk factors considered to predispose patients for PPD like smoking, and alcohol drinking were not documented. Seven (15.2%) patients had history of previous PPI use and one (2.2%) patient had history of previous eradication therapy. Forty-four (95.7%) had peritonitis at time of presentation.

The presenting complaints among the patients that had peritonitis at presentation were, forty-three (93.5%) diffuse abdominal pain thirty-eight (82.6%) vomiting, thirty (65.2%) epigastric pain and six (13 %) abdominal distension. Two patients (4.65%) presented with right lower quadrant pain and operated with the diagnosis of acute appendicitis, however, intraoperatively it was found to be perforated PUD. Only two types of tests were used (i.e. stool antigen test and serology) for all patients. All 46 patients were positive for either stool test or serologic test. Two of the patients were positive for both stool antigen and serology tests. Out of 46 patients, ten (21.7%) patients had positive result for stool antigen whereas eleven (23.9 %) patients had positive serology result for H. Pylori (Table 1 and 2). Overall, nineteen (41.3%) of patients tested positive for both H. Pylori stool antigen and serology tests.

**Table 1:** Stool antigen test results of patients operated for perforated PUD at SPHMMC from January 9, 2013 to February 7, 2018

Stool antigen test result	Frequency	Percent
Positive	10	21.7
Negative	17	37.0
Not done	19	41.3
Total	46	100

**Table 2:** Serology test result for patients operated for perforated PUD at SPHMMMC from January 9, 2013 to February 7, 2018

Serology test result	Frequency	Percent
Positive	11	23.9
Negative	13	28.3
Not done	22	47.8
Total	46	100

**Outcome**: Intraoperatively, forty-three (93.5%) patient had perforation on the first part of duodenum, one (2.2%) patient had gastric perforation and two (4.3%) patient had sealed perforation. For all patients with perforated peptic ulcer disease Graham's patch was performed. All patients had pre-operative stay of less than 24 hours. Twenty-three (50%) patient had postoperative stay of less than one week. Five (10.9%) patients stayed in the hospital greater than 2 weeks. During their hospital stay, majority of patients thirty-seven (84%) had no complications. five (10.9%) patients developed deep and superficial surgical site infection (Table 3).

**Table 3:** Postoperative complication after omental patch for patients operated for perforated PUD at SPHMMC from January 9, 2013 to February 7, 2018

Complication	Frequency	Percent
Deep and superficial	5	10.9
SSI	2	4.3
Leak	37	80.4
No	2	4.3
Not related to surgery	46	100
Total		

Forty (87%) were discharged improved and two (4.3%) died (Table 4).

**Table 4:** Outcome of the patients operated for perforated PUD at SPHMMC from January 9, 2013 to February 7, 2018

Outcome	Frequency	Percent
Discharged improved	40	87
Dead	2	4.3
Unknown	4	8.7
Total	46	100

Out of 46 patients, even though nineteen patients (41.3%) had positive results for H. Pylori test, twenty-six (56.5%) patients were given eradication therapy and thirty four (73.9%) patients were given PPI alone or as a part of eradication therapy (Table 5).

**Table 5:** Proportion of patients who were given eradication therapy after omental patch at SPHMMC from January 9, 2013 to February 7, 2018

Eradication	Frequency	Percent
Yes	26	56.5
No	9	19.6
Not documented	11	23.9
Total	46	100

## Discussion

In this study, the prevalence of H. Pylori among patients with PPU is comparable with findings with John B 46.9% and Young Joo Yang 44.8% (7, 15). But it was lower than the finding of others J Metzger 73.3% and 70% Saxena G (2, 3). Differing results in the diagnosis of H. Pylori were found in different studies based on the kind of test utilized. Sensitivity and specificity of serologic assay varies from 52–94% and 60–97 % respectively; on the other hand, biopsy-based methods have a low sensitivity but higher specificity (2, 3, 8). Besides serology and histopathology Urease (CLO) test is also used to diagnose H. Pylori in PPD, and was found to be 73.3% (2). In our study H. Pylori test was positive for stool antigen in 21.7% and for serology in 23.9 % of patients. Overall, 41.3% of patients had positive result regardless of the type of test used. This is comparable with study by the results of John B et al. 46.9% (7) and Young Joo Yang et al (15) 44.8%. In contrast serology results were positive in 100% of patient with PPD in two other studies but the histopathology yield after endoscopic biopsy dropped to 66.6% (3) and 70 %(18). Some of the factors for the variation of prevalence of H. Pylori in PPD could be different population group studied in unrelated geographic area (3) and different diagnostic modalities used for diagnosis having different sensitivity and specificity.

The Gender distribution was skewed to male, M: F ratio 6:1 similar to the study at Tikur Anbessa Hospital M: F ratio of 7:1 (20). In other studies, the gender distribution was similar except the mean age was older, 64 years, almost double the results of our study (2, 3, 4, 6, 22). The reason for older mean age in these studies could be the higher life expectancy in the developed world with older population compared to LMIC. Additionally, the risky behaviors of our younger male population (alcohol drinking, smoking and chat chewing) may contribute to male predominance.

There are conflicting data with regard to association of PPD to H. Pylori infection. Several studies showed socioeconomic status, prevalence of H. Pylori, smoking habits and alcohol are associated with or influence PPU rate (4, 16, 21, 22). On the other hand, there was no significant association between the incidence of H. Pylori infection in peptic ulcers with smoking and NSAIDS intake (7, 13, 18). To make these associations even more complex in one study it was found that PPD was associated with H. Pylori but not NSAID use (2). Unfortunately, in our study, there was incomplete documentation of risk factors in majority of patients and it was impossible to determine association.

Perforated peptic ulcer disease is a surgical emergency, patients usually present as an acute abdomen (15, 22). In this study 95.7% patients were diagnosed to have peritonitis with varying percentages of specific symptoms of peritonitis; diffuse abdominal pain 93.5%, vomiting 82.6% and epigastric pain 65.2%, which is similar to studies in Korea, Nigeria and other studies in Ethiopia (15, 17, 20). Intraoperatively, 93.5% patient had perforation on the first part of duodenum, which is similar to other African and Middle Eastern countries (22). On the contrary, the site of perforation in European studies has shifted from predominantly duodenal perforation to gastric perforation, where gastric perforation is now at 65% (22). A possible explanation for the variation in site of perforation according to several studies from developed countries is, lower socioeconomic development and H. Pylori infection are associated with PPD (2,4,16,21,22). Additionally, in the areas the improved socioeconomic status, use of PPIs and H. Pylori eradication treatment resulted in shift of perforation from duodenal to gastric perforation (22). In line with H. Pylori being associated with PPD, a study by Tokunaga Y et al (23) also found a higher density of H. Pylori with perforations, suggesting a potential dose effect relationship leading to perforation that can partly explain duodenal perforations in these geographical areas of low socioeconomic development (22). Additionally, the drop in the prevalence of H. Pylori in many western countries, coupled with increased use of NSAID in the elderly, resulted in the change from duodenal to gastric ulcers that potentially end up in perforation (24, 25).

For all our patients with PPD, Graham's patch was performed while on two patients with sealed perforation, only peritoneal lavage was done. Post operatively 56.5% of patients received H. Pylori eradication therapy even though only 41.3% of patients were positive for H. Pylori test which indicates that treatment was not based on the lab results. Studies that showed association of H. Pylori with PPD recommend simple closure of perforation (Graham's patch) should be followed by eradication treatment in all patients to prevent recurrence (2, 3,8,16,21, 22). On the other hand in a study that found no association suggested, instead of prescribing all patient with H. Pylori eradication treatment after Graham's patch, selective provision of eradication therapy for those who tested positive for H. Pylori, aimed at healing of the ulcer and decrease ulcer recurrence and reperforation is suggested (4).

The mortality rate of this study was 4.3% which is lower than other reports of 10–70% (6, 21,22).The reason for this could be the relatively younger patients in our study who got better physiologic reserve. They can also mount stronger immune response in the early phase of the disease and symptoms a signs will not bemasked thereby settling the diagnosis earlier in the course of the disease that facilitates early surgical intervention decreasing sepsis. In comparison delayed diagnosis, especially in the western world where obesity, comorbidity and immune compromised status of patients may mask clinical features delaying the diagnosis resulting in full blown peritonitis resulting in higher mortality due to sepsis (22).The main limitations of the study are incomplete documentation of risk factors on medical records, H. Pylori test was not done for all patients with PPD and lack of standardization of H. Pylori test with respect to which kind of test should be done. Despite these limitations the study assessed the prevalence H. Pylori of PPD in our hospital and an initial guide in the use H. Pylori eradication treatment in this group of patients.

In conclusion the prevalence of H. Pylori in PPD is relatively low, compared to most studies. H. Pylori eradication therapy should be based on the lab tests confirming its presence. A prospective study with good medical record keeping and, standardization of H. Pylori test will help in the understanding the association of H. Pylori with PPD and generate stronger recommendation on treatment of patients with PPD in our setting.
